# Diversity and Co-occurrence Pattern Analysis of Cecal Microbiota Establishment at the Onset of Solid Feeding in Young Rabbits

**DOI:** 10.3389/fmicb.2019.00973

**Published:** 2019-05-10

**Authors:** Tehya Read, Laurence Fortun-Lamothe, Géraldine Pascal, Malo Le Boulch, Laurent Cauquil, Beatrice Gabinaud, Carole Bannelier, Elodie Balmisse, Nicolas Destombes, Olivier Bouchez, Thierry Gidenne, Sylvie Combes

**Affiliations:** ^1^GenPhySE, Université de Toulouse, INRA, ENVT, Toulouse INP, Castanet Tolosan, France; ^2^Terrena, Ancenis, France; ^3^INRA, PECTOUL, Castanet Tolosan, France; ^4^INRA, GeT-PlaGe, Genotoul, Castanet Tolosan, France

**Keywords:** co-occurrence pattern, diversity, microbiota, age, weaning, onset of solid feeding, rabbit, cecum

## Abstract

This study aimed to evaluate how the feeding strategy of rabbit kits at the onset of solid feed intake could affect ecological diversity and co-occurrence patterns of the cecal bacterial community. From birth to 18 days of age kits were exclusively milk-fed, and between 18 and 35 days the young rabbits also had access to solid feed. After weaning at (35 days), young rabbits were exclusively fed solid feed. Three experimental feeds were used: a high concentrate diet [H: 10.16 MJ digestible energy (DE)/kg and 15.3% crude protein (CP)], a low concentrate diet (L: 9.33 MJ DE/kg and 14.7% CP) and a reproductive female diet (R: 10.57 MJ DE/kg and 17.3% CP). The rabbit kits (*n* = 357) were divided into three groups, differing by the diet received during two periods: from 18 to 28 and from 28 to 49 days of age. In the groups LL and HH, rabbit kits were fed L or H diets, respectively, during both periods. Kits in the group RL received feeds R and L from 18 to 28 and 28 to 49 days of age, respectively. Cecal bacterial communities of 10 rabbits per group were carried out at 18, 28, 35, 43 and 49 days of age by MiSeq Illumina sequencing 16S rRNA encoding genes. Between 18 and 28 days of age, solid feed intake was higher in the group RL compared to the other two groups (+24%; *P* < 0.01). Overall, 13.4% of the OTUs detected were present in the cecal ecosystem from 18 to 49 days old, whereas 17.4% were acquired with the onset of solid feeding and kept from 28 days on. Exclusive milk consumption constrains the bacterial community toward a similar structure but high phylogenetic beta-diversity. Introduction of solid feed induced a sharp change of microbial community structure and decreased phylogenetic diversity. A strong relationship in bacterial community network occurred only from 43 days on. Our feeding strategy at the onset of solid feed ingestion exhibited only a moderate effect on the microbial community structure (*P* = 0.072), although the LL group seemed to reach faster maturity compared to the two other groups.

## Introduction

The intestinal tracts of mammals are almost sterile at birth, where the installation and maturation of the bacterial communities are influenced by many factors, such as mode of delivery, milk source, the type of feeding and antibiotic therapy (Penders et al., 2006; Palmer et al., 2007; Koenig et al., 2011). The microbiota have a number of physiological roles, such as the hydrolysis and fermentation of nutrients, the regulation of the immune system and act as a barrier against infectious agents (Sommer and Backhed, 2013). As the colonization of the young mammal intestine by bacteria plays a major role in the development and refinement of host intestinal health and function, it is important to ensure a proper ecological succession of bacterial communities.

The transition from a milk-based diet to the ingestion of solid feed is often a critical period for mammals, where individuals undergo drastic nutritional changes. This transition period has a considerable impact on microbiota as the microbial community adapt to incoming solid feed with drastic changes in its composition and function (Mach et al., 2015). The rabbit is a good model to study the effect of feed transition on gut microbiota establishment due to its feeding behavior and because it is a polytocous species. After a short gestation time (31 days) rabbit kits are born naked, blind and only partly mature. Mastication is functional from 10 to 13 days of age (Langenbach et al., 2001) and milk fed rabbits start ingesting solid feed far before weaning (around 35 days of age). Unlike other altricial species, the rabbit female nurses it’s young only once a day (Coureaud et al., 2000) and then leaves the nest. This specificity allows for the easy control of the quantity of milk made available to the rabbits. From birth to weaning, rabbit kits change from a milk rich in protein and fat (12.3 and 12.9 g/100 g, respectively, Maertens et al., 2006) to a feed that is rich in starch and vegetal protein, and poor in fat (140, 125 and 30 g/kg, respectively, Gidenne and Fortun-Lamothe, 2002). This drastic nutritional change coincides with a high occurrence of digestive problems and mortality rate. There are two hypotheses to explain the digestive distortion, both based on an incompatibility between the young rabbit digestive maturity and the composition of feed ingested. Firstly, at the onset of solid feed ingestion, the rabbit kit digestive system is insufficiently mature, notably the level of enzymatic digestion in the proximal part of the digestive tract, to ensure complete digestion of the feed (Debray et al., 2002). Previous studies noted that the rabbit digestive system is at full capacity around 45 to 50 days of age, one to two weeks after weaning (Debray et al., 2002). Secondly, the feeds distributed to rabbit kits may not be adapted to favor the installation of a proper bacterial community able to stimulate the immune system and improve animal health. Currently, the mother and their kits have access to the same feed before weaning through they have antagonistic nutritional needs (Gidenne and Fortun-Lamothe, 2002). Females have high energy needs mostly supplied through high dietary starch levels, while previous studies reported an inverse relationship between fiber and starch has had favorable effects on the incidence of diarrhea in rabbit kits (Gidenne, 2015). The introduction of feed better adapted to physiological stage of animals could improve the health of rabbit kits.

Intake of solid feed conditions the development of the microbiota. When young rabbits were subjected to an exclusively milk-based diet until weaning, development of the cecum, pectinolytic and xylanolytic activity were lower and the biodiversity index was lower at 30 days than in control animals (Combes et al., 2008). Furthermore, exclusive ingestion of milk appears to delay colonization by cellulolytic bacteria without affecting the population of *Escherichia coli* (Padilha et al., 1995, 1999). On the contrary, when access to milk is stopped through early weaning, the solid feed intake is stimulated as well the cecal microbial activity (Gallois et al., 2008), but rabbit health is impaired (Feugier et al., 2006). This is probably due to the lack of passive immunity via the milk (Gallois et al., 2007).

The introduction of solid feed is a key step in the development of intestinal microbiota and young rabbit specific feed has to be designed to stimulate solid feed ingestion and promote the maturation of the bacterial community. Read et al. (2015, 2016) have previously shown that the feeding strategy at the onset of solid feed intake influences the early feed ingestion, where feed high in energy had a positive effect on early feed intake (Read et al., 2015, 2016), health and growth (Read et al., 2015). In this study, we compared the common rabbit feeding strategy in which young rabbits have the same feed as their mothers until the age of 28 days with a strategy of introducing from 18 days of age either a high concentrate or low concentrate feed that will be offered to young rabbits after weaning. However, to our knowledge, the impact of the development of solid feed intake on the microbiota development of kits is not known. Therefore, this study aimed to determine how the feeding strategy at the onset of solid feed intake of milk-fed rabbits could affect ecological diversity and co-occurrence patterns in cecal bacterial community in young rabbits.

## Materials and Methods

### Ethics Statement

This study was carried out in accordance with the European Union recommendations on the protection of animals used for scientific purposes at the PECTOUL Experimental Unit (INRA, Toulouse, France), and was approved by the French Government (No. 2015100817517471).

### Animals, Diet and Experimental Design

The animals used in the experiment were a crossbreed from maternal line INRA 1777 and paternal line Hyplus. They were raised in an experimental farm that kept standard commercial handling conditions. Cages were equipped to study feed intake of the kits independently from their mother (Fortun-Lamothe et al., 2000). Lactation lasted 35 day. At weaning litters were split into cages of 5 kits/cage in the fattening area, in function of weight ranks within the litter.

A total of 537 rabbit kits were used in this study. The animals were distributed at birth into one of three experimental groups depending on their mothers’ weight at parturition (4254 ± 392 g) and parity (4.7 ± 3.5), as well as the litter size at birth (11 ± 3 kits). The litters (*n* = 56) were equalized at 10 kits, 3 days after birth (day 0) by cross-fostering or culling. Doe milk production between birth and day 21 of lactation was calculated for the three experimental groups according to Fortun-Lamothe and Sabater (2003). All 56 reproductive females were fed the reproductive female diet [R diet 10.57 MJ DE, 173 g CP/kg, 166g starch/kg and 173 g acid detergent fiber (ADF)/kg; Table 1 and Supplementary Figure S1] *ad libitum* throughout the study. This diet has been formulated to meet the nutrient requirements of reproductive females (De Blas and Mateos, 1998). Experimental groups differed by the diet received by kits during two different periods: from 18 to 28 days of age, and from 28 days of age until the end of the study (Supplementary Figure S2). In the group LL, the kits received a low concentrate diet (the L diet, 9.33 MJ DE/kg, 147g CP/kg, > 224g ADF) during both periods. The group HH kits received a high concentrate diet (the H diet, 10.16 MJ DE/kg, 153g CP/kg, > 204g ADF/kg) during both periods. Both the H and L diets were formulated to meet the nutrient needs of growing rabbits as they are poor in starch (<120 g/kg) and rich in fiber (ADF > 204 g/kg) to prevent digestive troubles (Gidenne, 2015). The third group, RL, received the R diet, same as that offered to females, from 18 until 28 days of age, and the L diet was distributed from 28 days of age until the end of the study. This feeding strategy is close to current practices in French commercial rabbit farms whilst LL and HH feeding strategies were specifically designed for the young rabbits. We hypothesized that these latter would be better adapted to promote health and growth of the young rabbits. Between 18 days of age and weaning (35 days of age), animals were fed *ad libitum*. After weaning, feed was restricted in function of a predefined computation grid according to the average weight of the group at weaning. The experiment took place from 18 to 49 days of age, and corresponds to the distribution of experimental solid feed. At 18, 28, 35, 42 and 49 days of age, ten rabbits from each treatment were individually weighed, then slaughtered by electronarcosis and exsanguination. Animals slaughtered were representative of their litter (before weaning at 35 days of age), or their cage (after weaning), and in good health. The cecum was isolated and samples of the cecal content were collected and stored at −80°C. The pH (Unitrode with Pt 1000; Metrohm, Herisau, Switzerland) was recorded and two samples (1 g each) of fresh cecal content were diluted in storage solutions, 1 in HgCl_2_ (2 mL, 2% w/v) and 1 in H_2_SO_4_ (3 mL, 2% w/v), for further analysis of volatile fatty acids (VFA) and NH_3_, respectively. Quantifications of VFA were performed by automated GC (Chrompack CP 9000; Chrompack B.V., Middelburg, Netherlands) according to Playne (1985). The NH_3_ concentrations were determined using a colorimetric method by a continuous flow analyzer (SAN11; Skalar, Norcross, GA, United States) as described previously (Verdouw et al., 1977). Dry matter (DM) was determined in cecal samples by drying at 103°C for 24 h.

**Table 1:** Chemical composition of experimental diets.

Chemical composition (g/kg)	Diet R^a^	Diet L^b^	Diet H^c^
Crude Protein	173	147	153
Crude Fat	31	26	29
Crude Cellulose	143	179	164
Starch	166	70	113
Sugars	58	0	0
Ash	74	90	85
Acid detergent fiber (ADF)	173	220	200
Neutral detergent fiber (NDF)	320	390	351
Acid detergent lignin (ADL)	56	69	58
Digestible fiber	199	258	240
Digestible energy (MJ/kg; DE)	10.57	9.33	10.16
Digestible protein (DP)	128	98	106
DP/DE	12.1	10.5	10.4

*^a^ R (Reproduction diet): diet formulated to meet the needs of reproductive females. ^b^ L (Low concentrate diet) and ^c^ H (High concentrate diet): diets formulated to meet the needs of growing rabbits with differing energy and protein levels, and a constant DP/DE ratio.*

### DNA Extraction and PCR Amplification of Bacterial 16S Ribosomal Genes for MiSeq Illumina Sequencing

Total genomic DNA was extracted from samples of cecal content and purified using the QIAamp^®^ DNA Stool Mini kit (Qiagen Ltd., West Sussex, United Kingdom) according to the manufacturer’s instructions after thermal (from −80°C to 95°C) and mechanical lyses (bead beating using 400 mg of 0.1 mm glass beads). The quality and quantity of DNA extracts were observed using a spectrophotometer (ND-1000; NanoDrop Technologies, Wilmington, DE, United States).

The V3-V4 regions of 16S rRNA genes of samples were amplified from purified genomic DNA with the primers F343 (5′–CTTTCCCTACACGACGCTCTTCCGATCTACGGRAGGCAGCAG –3′; (Liu et al., 2007) and reverse R784 (5′–GGAGTTCAGACGTGTGCTCTTCCGATCTTACCAGGGTATCTAATCCT–3′; (Andersson et al., 2008). The PCR was carried out with an annealing temperature of 65°C for 30 amplification cycles to minimize PCR biases. As MiSeq enables paired 250-bp reads, the ends of each read are overlapped and can be stitched together to generate high-quality, full-length reads of the entire V3 and V4 region in a single run. Single multiplexing was performed using 6 bp index, which were added to R784 during a second PCR with 12 cycles using forward primer (AATGATACGGCGACCACCGAGATCTACACTCTTTCCCTACACGAC) and reverse primer (CAAGCAGAAGACGGCATACGAGATGTGACTGGAGTTCAGACGTGT). The resulting PCR products were purified and loaded onto the Illumina MiSeq cartridge (Illumina, San Diego, CA, United States) at the Genomic and Transcriptomic Platform (INRA, Toulouse, France) according to the manufacturers’ instructions. Each pair-end sequence was assigned to its sample with the help of the previously integrated index. Sequencing reads were deposited in the National Center for Biotechnology Information Sequence Read Archive (SRA accession: SRP072123).

### Sequence Analysis

A total of 10,612,613 16S ribosomal DNA amplicon sequences were cleaned, clustered in OTU (operational taxonomic unit) and affiliated to taxa using the FROGS pipeline (Escudié et al., 2018). Using FROGS, in keeping with the SOP, sequences were filtered by removing sequences that did not match both proximal PCR primer sequences (no mismatch allowed), erroneous sequencing length (<400 or >500 nucleotides), with at least one ambiguous base. Chimeric DNA sequences were detected using VSEARCH (Rognes et al., 2016) and removed. A total of 7,300,458 reads were retained corresponding to 48,670 ± 11,497 reads per sample. Reads were clustered into OTUs using SWARM (Mahé et al., 2014). OTU taxonomic assignment was performed using the BLAST algorithm (Edgar, 2010) against the SILVA SSU Ref NR 132 database (Quast et al., 2013). A phyloseq R package (McMurdie and Holmes, 2013) object was generated and the Shannon and the InvSimpson diversity indices were calculated. Using the function “taxglom” from the “phyloseq” R package on the OTUs dataset, three taxonomical datasets were generated for phylum, family and genus levels, respectively.

### Statistical Analysis

All statistical analyses were carried out using R version 3.3.2. Cecal fermentation parameters, number of observed OTUs, the diversity indices, and after taxonomical assignment, the relative abundances at phylum, family and genus levels were analyzed using a linear mixed model with group and age as fixed effects and the doe as random effect. A Benjamini Hochberg multiple test correction was applied. Group averages were then compared using the Tukey test. The dissimilarity between the bacterial communities was represented by nMDS (non-Metric Dimensional Scaling) using both the Bray-Curtis and UniFrac distance calculations on the rarefied count matrix (R package vegan). Bacterial community maturity was evaluated according to distance calculations to reach the 49-day-old rabbit community. Distances were calculated within the corresponding diet group for each sample at a given age relative to all the samples at 49 days of age using “dist_group” function from “usedist” R package. Homogeneity of dispersion among groups was tested using “betadisper” function (R package vegan). To check group differences an ADONIS pairwise test with the Bray-Curtis distance was carried out.

Network analysis was used to explore co-occurrence patterns of microbial OTUs within an age group. The OTUs with abundances above 1% of the rarefied count matrix were selected. A Spearman’s correlation between two OTU count values was considered if the Spearman’s correlation coefficient (ρ) was > 0.7 thus resulting in a maximum *P*-value of < 2.32e-05 (Williams et al., 2014). A correlation network was built (R package igraph) (Csardi and Nepusz, 2006), where each node represents one OTU and each edge stands for correlation between the OTU abundances. Metrics to describe the resulting networks were numbers of nodes and edges, mean degree and normalized mean degree, and betweenness centrality. Finally, intersection graphs were built to test persistence of co-occurrence pattern across two consecutive ages. The functional potential of main taxa was explored using MACADAM database (MetAboliC pAthways Database for Microbial taxonomic groups) (Le Boulch et al., 2019), and its associated script: MACADAMExplore^1^. Briefly, MACADAMExplore is a python script facilitating the queries on MACADAM database to get taxonomic functional information. All the metabolic pathways present in a taxa is listed and each pathway is associated to a metabolic pathway completeness score. For a given taxon, the percentage of organisms that contain the metabolic pathway is calculated. A pathway is kept if it is present in at least 33% of organisms of the taxa of interest. Feed intake was analyzed by a linear mixed model, taking into consideration the group as a fixed effect, the doe as a random effect and the weight at equalization as a covariable (R package lmer).

## Results

### Milk and Feed Intake

Animals used in this study were in good health (mortality rate: 2.4% between 18 and 49 days of age for all groups). Average doe milk production was similar between groups (3962 g between birth and 21 days of age, *P* = 0.163). At 18 days, kits weighed 297 ± 50 g, 309 ± 52 g and 309 ± 49 g for RL, LL, and HH group, respectively (*p* = 0.026, Supplementary Table S1). Average feed intake of the kits between 18 and 28 days of age was 6.8 g/d/kit. During this period, feed intake was about 24% higher in the RL group compared to the LL and HH group (Table 2; *P* = 0.032). Average feed intake during the period of 28 to 35 days of age was 19 g/d/kit for all three groups (*P* = 0.392). During the period of feed restriction, from 35 to 49 days of age, no remaining feed was found in the feeder, therefore feed intake corresponded to feed offered.

**Table 2:** Solid feed intake of rabbits before and after weaning (35 days) according to the feeding strategy.

Groups	RL	LL	HH	SEM	*P* value
No. animals	171	189	177		
**Feed intake (g/kit/day)**					
18–28	7.8^a^	6.4^b^	6.2^b^	0.11	0.032
28–35	19	21	16	0.51	0.392
35–42	70	60	65	nc^1^	nc
42–49	95	85	85	nc	nc

*^1^ nc: not calculable since ingestion was limited and no remaining feed was found in the feeder, therefore feed intake corresponded to feed offered. ^a,b^: Means with different superscripts differ at P < 0.05.*

**Table 3:** Age-related changes in bacterial alpha-diversity for young rabbit cecum (mean ± standard deviation).

Age (days)	Number of observed OTU	Shannon index	InvSimpson index
18	229 ± 38 ^a^	3.28 ± 0.13 ^a^	13.6 ± 2.4^a^
28	425 ± 61 ^b^	4.39 ± 0.32 ^b^	38.3 ± 16.2^b^
35	475 ± 72 ^c^	4.53 ± 0.37 ^b^	41.5 ± 18.1^b^
43	492 ± 68 ^cd^	4.52 ± 0.39 ^b^	40.1 ± 19.5^b^
49	518 ± 55 ^d^	4.72 ± 0.26 ^c^	46.6 ± 18.6^b^
***P* value**			
Age	<0.001	<0.001	<0.001
Diet Group	0.170	0.578	0.767
Age^∗^ Diet group	0.096	0.016	0.053

*Means with different superscripts differ at P < 0.05.*

### Diversity and Structure Dynamics of the Cecal Bacterial Community

**FIGURE 1:**
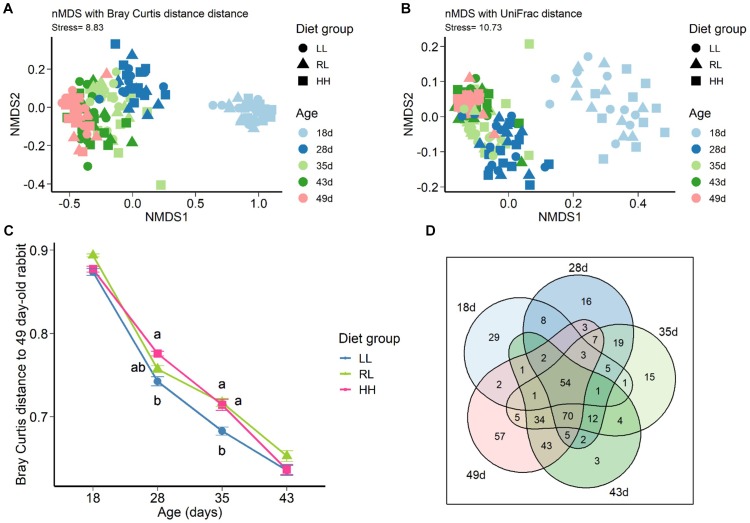
Non-Metric Dimensional Scaling (nMDS) two-dimensional representation of cecal bacterial community using Bray Curtis **(A)** and Unifrac distances **(B)** of rabbits in groups LL, RL, and HH from 18 to 49 days of age. Maturity of cecal bacterial community **(C)** the higher the value, the greater the dissimilarity of the bacterial community is from 49 days; means with different superscript differ at *P* < 0.05. Venn diagram **(D)**: occurrence of the OTUs across the cecal ecosystems from 18 to 49-day-old. The OTUs detected in at least 75% of the samples of the same age group were conserved.

Age-related changes and feeding strategy at the onset solid feed intake on cecal bacterial community diversity was first assessed using the Shannon and InvSimpson indexes (Table 3). As expected, the number of OTUs observed and diversity increased with age (*P* < 0.001), but no effect of feeding strategy was observed at any ages. A Venn diagram was used to display the numbers of common and unique OTUs present in 75% of individuals per age group (Figure 1D), out the 402 OTUs, 13.4% OTUs were present in cecal ecosystem from 18 days to 49 days old, whereas 29 OTUs were present only in milk-fed rabbits and 17.4% (70 OTUs) were acquired with the onset of solid feed intake and were present until 49 days of age. The latter were assigned mainly to Lachnospiraceae and Ruminococaceae families (34 and 27 out of 70 OTUs, respectively). Among known genus, bacterial OTUs exclusively inhabiting the 75% of milk-fed rabbit cecum belong to *Bacteroides* (25%), dgA-11 gut group (12%), *Alistipes* (4%). Finally, exclusively milk-fed rabbits shared 64 OTUs (16%) with their exclusively plant-based, solid feed 43 and 49-day-old counterparts. These OTUs represent a relative abundance of 80.1, 27.7, and 20.6% of the community at 18, 43, and 49 days, respectively (Supplementary Figure S3).

The nMDS plot indicated that both the structure (Bray Curtis distance calculation, Figure 1A) and composition (unweighted UniFrac distance calculation, Figure 1B) of the bacterial communities changed according to age. The exclusively milk-fed 18-day-old bacterial community was separate from the communities of the other age groups. A milk exclusive diet lead to strong homogenous intra age group structure community according to Bray Curtis distance (multivariate homogeneity of 18 day group dispersion against all the other age groups *P* < 0.05 Supplementary Figure S4). However, according to UniFrac distance calculation, a high taxonomic variability is observed at the same time (Supplementary Figure S4). ADONIS performed on the Bray Curtis distance matrix confirm only a moderate effect of diet group (*P* = 0.072) on microbiota shaping. Considering distance to reach 49-day-old community structure as a maturity indicator of the ecosystem (Figure 1C), a highest maturity could be observed in cecum microbiota of the LL group rabbits whose community structure was closest to the 49-day-old one compared to the two other groups (*P* < 0.05).

### Dynamics of the Taxonomical Composition of the Cecal Bacterial Community

**FIGURE 2:**
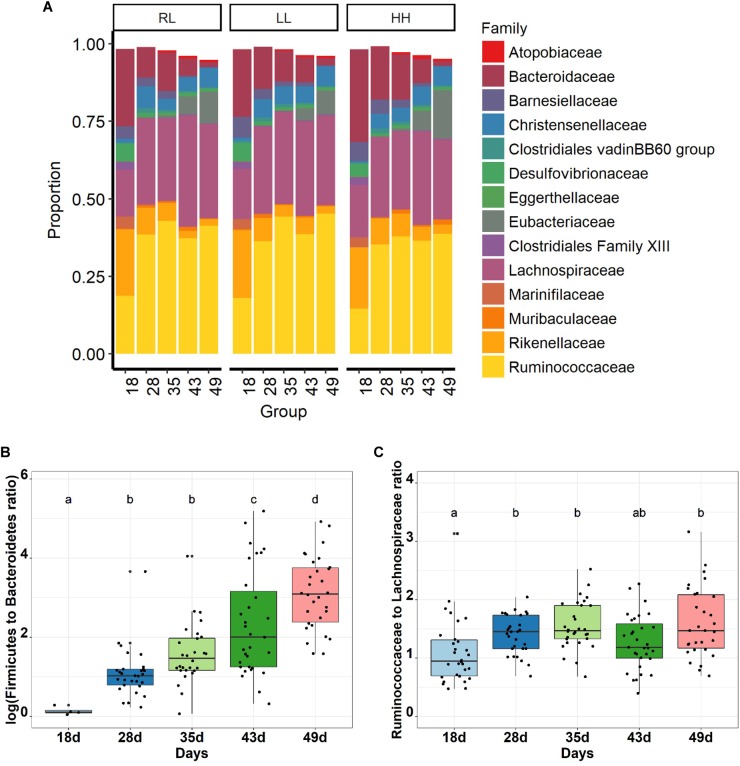
Age related bacterial family distribution for the three experimental groups **(A)**, and age related change of Fimicutes to Bacteroides ratio **(B)** and Ruminococcaceae to Lachnospiraceae ratio **(C)** in rabbit cecal bacterial communities (*n* = 30 rabbits per age).

As expected, Firmicutes was the dominant phyla (72%) followed by Bacteroidetes (24%) and Proteobacteria (2%), while Actinobacteria and Tenericutes represented less than 1% (Supplementary Table S2). Based on a BLAST 97% identity threshold, 98.8 and 74.0% of the sequences could be assigned at the family and genus (or genus group) levels, respectively, and 1.2% of the sequence had a multi affiliation for the genus. Ruminococcaceae (35%), Lachnospiraceae (26%), Christensenellaceae (5%) and Eubacteriaceae (4%) were the most abundant families belonging to the Firmicutes phylum (Figure 2A). Bacteroidaceae (12%), Rikenellaceae (8%) and Barnesiellaceae (2%) were the most abundant families accounting for the Bacteroidetes phylum. A total of 72 genera, or genus groups, were found in the cecum of healthy rabbits, from which *Bacteroides*, Ruminococcaceae NK4A214 group and Lachnospiraceae NK4A136 group were the most predominant, representing 11, 7.8 and 7.0%, respectively, of the total abundance (Supplementary Figure S5 and Supplementary Table S4). The taxonomical profiles of the cecal bacterial community revealed an age-related change in the taxa relative abundance (*P* < 0.001, Figure 2 and Supplementary Tables S2–S4). The Bacteroidetes phylum was dominant at 18 days of age with a relative abundance of 56%, which then sharply decreased to 6% at 49 days of age (*P* < 0.001; Figure 2B; Supplementary Table S2). Concurrently, the Firmicutes phylum increased from 18 until 49 days of age (37 to 91%, *P* < 0.001) becoming dominant over the Bacteroidetes as early as 28 days of age. The Firmicutes to Bacteroidetes ratio rose from 0.65 ± 0.23 to 33 ± 32 (*P* < 0.001, Figure 2B). Proteobacteria represented around 5% at 18 days of age and the abundance decreased 3-fold at 49 days of age (*P* < 0.001). All families were affected by age, yet two families (Ruminococcaceae and Lachnospiraceae) were stable after 28–49 days of age (Figure 2C and Supplementary Table S3). The Ruminococcaceae family became dominant over Lachnospiraceae in the ecosystem from 28 days on.

### Cecal Fermentative Parameters

All of the cecal fermentation parameters were affected by age (Supplementary Tables S5, S6). The proportion of butyrate increased and the ratio propionate to butyrate (C3/C4) decreased with age, as expected, while the total VFA concentration was found to increase from 18 to 35 days of age. From 42 to 49 days of age, the VFA observed in restricted fed animals was 30.2 mM/L. Experimental group had an effect on NH_3_ content, and an interaction age^∗^group was observed. The NH_3_ was +42% higher, respectively, at 28 days of age in the RL group compared to the two other groups (*P* < 0.01). The NH_3_ levels at 35 days of age were found to be higher in the RL group compared to the LL group, with the HH group as intermediary (*P* < 0.01).

**Table 4:** Co-occurring bacterial OTUs network metrics based on spearman correlation analysis in cecum microbiota for 18, 28, 35, 43, and 49-day-old rabbits.

Age (days)	Nodes	Edges	Normalized Degree	Mean degree	Betweenness	Diameter
18	31	29	0.070	1.87 ± 1.14	0.0043	1.67
28	31	20	0.057	1.29 ± 0.52	0.0068	1.49
35	51	35	0.032	1.37 ± 0.63	0.0084	2.90
43	89	175	0.160	3.93 ± 4.66	0.0190	4.54
49	72	180	0.211	5.00 ± 6.04	0.0342	3.62

### Co-occurrence Networks of Cecal Microbiota

**FIGURE 3:**
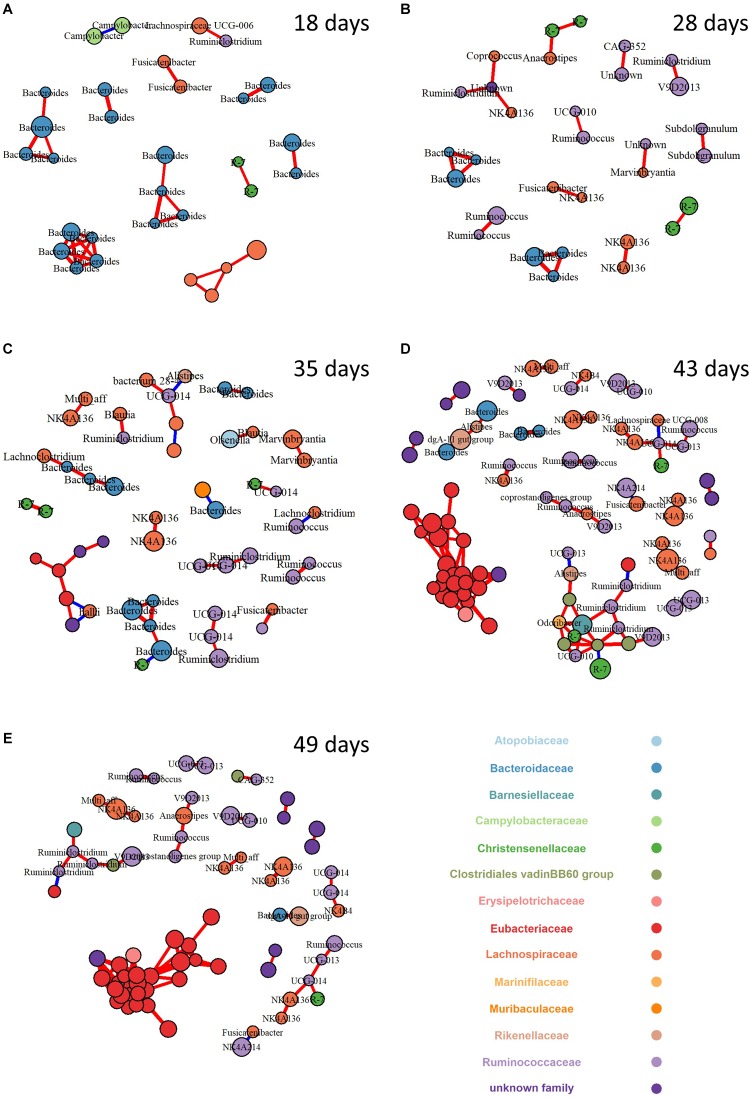
Network of co-occurring bacterial OTUs based on spearman correlation analysis in cecum microbiota for 18 **(A)**, 28 **(B)**, 35 **(C)**, 43 **(D)**, and 49 **(E)** day-old rabbits. A connection stands for a strong (Spearman’s ρ > 0.7) and significant (*P* < 0.0001) correlation. The size of each node is proportional to the OTU relative abundance, only OTU with a relative abundance higher than 1% were kept; the red and blue colors of each connection between two nodes (edge) stands for positive or negative correlations. The nodes (OTUs) were colored by family and labeled by genus when available.

To explore the change of bacterial interaction and keystone species in the ecosystems across ages, a bacterial community network analysis was performed for each age (Figure 3). Only strong connections (spearman correlation coefficient above 0.7) between OTU with relative abundance above (1%) were investigated. The number of nodes and edges increased with age with a 3 and 5 fold increase between weaning to 43 days of age, respectively (Table 4). Accordingly, mean degree and the normalized degree score, which allows for the comparison of the number of connections per node independently of the number of nodes in the network, sharply increased at 43 days and were at a maximum at 49 days of age. Finally, strength of betweenness centrality of network increased with age, which relates more complex inter-relationships of bacterial OTUs with aging.

To identify highly connected OTUs that may represent keystone species within their ecosystem, the individual-level metrics for each node across the networks was calculated (Supplementary Figure S6). At 18 days of age, OTUs assigned to *Bacteroides* set a central position while those assigned to Lachnospiraceae were already present. At 28 days of age, *Bacteroides* supremacy in the network declined in favor of an OTU assigned to an unknown genus from Rhodospirillales order (BLAST identity score of 93.3) and, to a lesser extent, to Christensenellaceae OTUs. At the same time, OTUs belonging to Ruminococaceae family appear in the network. From 35 days old onward, Eubacteriaceae OTU occupy an increasingly central position.

Intersections between networks from different ecosystems were also used to determine if any co-occurrence relationships were consistent across ecosystems of consecutive ages (Supplementary Figure S7). No common co-occurrence pattern could be observed between 18 and 28 days of age. Between 28 and 35 days of age, only one module linking three *Bacteroides* OTUs were consistent across ecosystems. Between 35 and 43 days of age, the intersection graph contained 11 nodes and 12 edges. Finally, the network resulting from intersections between 43 and 49-day-old networks was the most complex with more than 67 nodes and 148 edges in common which shows a conservation rate of 75 and 84% of the nodes and the edges, respectively, compared to the 43-day-old network. The 43–49 day intersection network exhibited a mean degree (4.4 ± 5.2) and normalized degree (0.206), that indicate that the most complex modules were likely conserved between the two ages.

**FIGURE 4:**
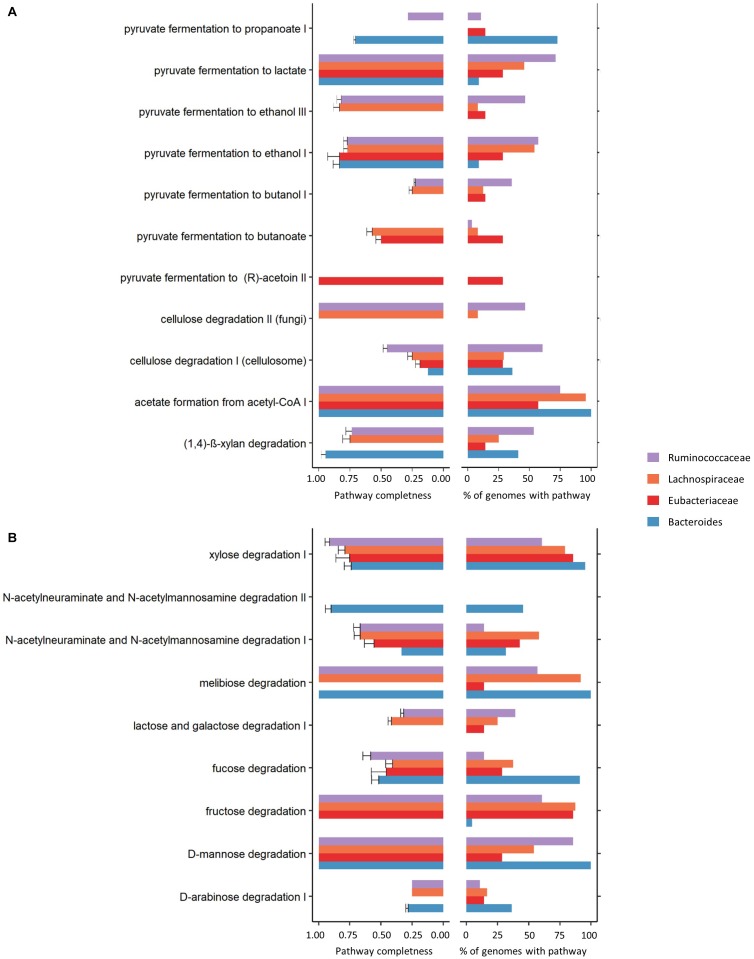
Breakdown of carbohydrate **(A)** and polymeric compound degradation and fermentation **(B)** showing completeness of the pathways (left) and their percentages of presence in genomes (right) in Lachnospiraceae (*n* = 24 genomes), Ruminococcaceae (*n* = 28 genomes) and Eubacteriaceae (*n* = 7 genomes) families and *Bacteroides* genus (*n* = 22 genomes).

Using MACADAM database ^2^ (Le Boulch et al., 2019; Figure 4 and Supplementary Figure S8), the functional potential was explored and, in particular, common and specific metabolic pathways performed by the main three families (Lachnospiraceae, Ruminococcaceae and Eubacteriaceae) and the main known genus (*Bacteroides*) that make up the networks. Four carbohydrate degradation pathways were identified in 90 to 100% of the 22 complete, high quality, annotated *Bacteroides* genomes (fucose, xylose, D-mannose and melibiose degradation). Interestingly, compared to the three other dominant groups in the ecosystem, *Bacteroides* was the only genus to be able to achieve the whole the *N*-acetylneuraminate and *N*-acetylmannosamine degradation II pathway (completeness score = 1, and present in 45% of the annotated genomes). Contrarily, Ruminococcaceae family members seem to be more highly specialized for pyruvate to lactate fermentation and for the degradation of complex plant material such as cellulose degradation. Lachnospiraceae had an intermediate metabolic position sharing carbohydrate degradation pathways and fermentation processes with *Bacteroides* (melibiose degradation, acetate formation from acetyl CoA, and xylose degradation) or with Eubacteriaceae (fructose degradation pathway). Eubacteriaceae bacteria exhibit certain metabolic specificities, including the absence of certain pathways such as (D-arabinose degradation I, lactose and galactose degradation I, melibiose degradation and (1,4)-β-xylan degradation) and the presence of four pathways of degradation of pyruvate.

## Discussion

The identification of digestive ecosystem bacterial communities and its maturation has been the topic of much research in a number of species, including rabbits (Padilha et al., 1995; Kovács et al., 2008; Monteils et al., 2008; Michelland et al., 2010; Combes et al., 2011; Bauerl et al., 2014; Jin et al., 2018). The digestive ecosystem plays an important role in the development and stimulation of intestinal immune system (GALT) (Thaiss et al., 2016), particularly in rabbits where both the GALT and humoral immunity development is dependent on specific intestinal microbiota stimulation (Rhee et al., 2004; Mage et al., 2006). Thus, in order to preserve animal health, attempts are performed to control implantation dynamics of the bacterial communities. Diet is one of the best tools to shape bacterial communities. Two lines of research can be envisaged, the first consists in proposing a food to stimulate an early solid ingestion and the second consists in seeking its optimal nutritional composition which would favor the installation and the maturation of the intestinal microbiota. The current study compares the common feeding strategy used in rabbit farm with two feeding strategies using either a high concentrate or low concentrate feed. We hypothesized that these nutritional modulations at the onset of feed intake in young rabbits would promote early solid feed ingestion and alter microbiota establishment. The current work describes in rabbits (1) diversity and co-occurrence patterns during the establishment of the bacterial community (2) and how feeding strategy at the onset of solid feed intake might drive the bacterial community composition and maturation.

### Cecal Bacterial Communities in Exclusively Milk-Fed Rabbits

After birth and all along the lifespan, microbiota undergoes constant evolution according to host physiological state, biotic and abiotic factors of host surroundings and diet. Considering the latter, the transition from milk to solid feed is one of the most influential factors driving microbial community composition (Mach et al., 2015). Consistently, our results showed that the bacterial communities evolve with age, showing a gradual increase in alpha-diversity and in bacterial relationship network complexity over time, however, the change from a strictly milk based diet to solid feed had the most marked effect. Before the introduction of solid feed, exclusive milk consumption constrains the bacterial community to a highly homogenous community structure. Besides the crucial nutritional value of the milk for the newborn, milk provides an important variety of compounds providing substrate for microbe development as prebiotic, but also antimicrobial and bacteriostatic (Hamosh, 1998), whose ingestion drives bacterial community structure. Rabbit milk contains mainly immunoglobulin G (95%) and A (2%), and is particularly rich in short chain fatty acid such as caprylic (C8:0) and capric acid (C10:0) together with transferrin (Baranyi et al., 1995; Maertens et al., 2006), which have antimicrobial or bacteriostatic effects. Additionally, rabbit milk contains glycoprotein carrying specific epitopes that are receptors for *Helicobacter pylori*, enteropathogenic and enterotoxic *E. coli* (Gustafsson et al., 2005). As suggested by Urashima et al. (2001), extremely low levels of lactose in milk, which is a characteristic of altricial species, such as marsupials and, to a lesser extent, rabbits, might suggest an important role of milk oligosaccharides on both commensal bacteria establishment and host development itself, either directly or in interaction. In this study, a milk exclusive diet lead to a hegemony of the *Bacteroides* genus. These hegemony might be linked to the habitat defined by milk derived abiotic factors Additionally, co-occurrence pattern analysis of cecal communities within exclusively milk-fed rabbits clearly demonstrate *Bacteroides* central position supremacy in the network. Interestingly, functional inference analysis based on available high quality annotated genome suggested not only the ability of *Bacteroides* to break down milk carbohydrates (e.g., fucose), but also the forthcoming plant derived substrates. The species of this genus are also able to produce sialic acid (*N*-acetylneuraminic) that plays important roles in their colonization process. However these functional inference analysis are limited to a subset of *Bacteroides* species with available annotated genomes and the presence of these pathways in rabbit cecum has to be further confirmed.

Notably during this milk exclusive period, concomitantly to the homogenous structure of the bacterial community, using Unifrac distance, we observed the greatest variability in the phylogenetic composition. Consistent with previous results observed in rabbits (Combes et al., 2011) and in pigs (Thompson et al., 2008), this bacterial phylogenetic diversity among individual milk-fed animals is a consequence of the random acquisition of microbes from large metacommunities, such as contact with the mother and the immediate surroundings. A concrete example of large microbial metacommunities in contact with rabbits is the fecal pellet deposed in the nest by the mother during nursing and eaten by the kits (Kovács et al., 2006; Combes et al., 2014). Accordingly, the exclusively milk-fed rabbits shared 16% common OTUs with exclusively plant-based fed rabbits. Additionally, in the cecal ecosystem from milk-fed rabbits, we could pinpoint Lachnospiraceae OTU assemblages occupying the second-most important position in the interaction network, after *Bacteroides*. This phylogenetic diversity is consistent with previous studies which reported the presence of microbial enzymes that degrade non digestible polysaccharides of plant origin in the gut of infants fed exclusively breast-milk (Koenig et al., 2011).

### Impact of the Onset of Solid Feed Intake on Microbiota Diversity

Once solid feed ingestion was initiated, structure and phylogenetic composition continue to change with age but less markedly than between 18 and 28 days. Reported results as to the age of stabilization of the cecal community in rabbits varies. Rodriguez-Romero et al. (2013) determined that the bacterial microbiota stabilized around 25–28 days of age, while Combes et al. (2011) observed significant fluctuations of the rabbit bacterial community composition until 49 days of age. Considering that the rabbit cecal microbiota is characterized by an overwhelming number of species belonging to the Fimicutes phyla, the Firmicutes to Bacteroidetes ratio could be viewed as a maturity index of the rabbit ecosystem. As seen in the present study, the Firmicutes to Bacteroidetes ratio increased until 49 days of age. According to microbial co-occurrence network analysis, the complexity of the network only occurs when solid feed ingestion is well established e.g., one week after weaning. At this time, numerous OTUs are assembled into consortia of interacting species. Moreover, the most complex co-occurrence relationships were consistent across cecal ecosystems one week later. After weaning, although not dominant in the ecosystem, Eubacteriaceae OTUs occupy a central position in the ecosystem interaction network having the highest degree number and these OTUs were mainly connected to each other. Meanwhile, OTUs belonging to Ruminococcaceae and Lachnospiraceae co-occurred together and with other phylogenetic taxa. According to Williams et al. (2014), microbial assemblage that co-occurs may share similar ecological characteristics. Compare to Lachnospiraceae and Ruminococcaceae, Eubacteriaceae bacteria exhibit certain metabolic specificities, including the absence of certain pathways such as (D-arabinose degradation I, lactose and galactose degradation I, melibiose degradation and (1,4)-β-xylan degradation). However, these results should be taken with caution as only a limited number of high quality annotated complete genomes could be used in this metabolic pathway inference analysis. The lack of knowledge on bacterial species inhabiting the rabbit cecum remains an important barrier that could only be overcome by effort in further sequencing and annotation of bacterial genomes.

Considering that the amount of feed consumed by rabbit kits when they first start ingesting solid feed had an influence on the fermentation parameters at weaning (Nizza et al., 2010), we suggest that stimulating at early age solid feed ingestion would be an efficient lever to drive gut microbial community establishment. In our study the highest feed intake at the onset of solid feed ingestion was observed in kits that received the same diet as that offered to females. Young animals before weaning might have an attraction for the feed distributed to rabbit does. Once the animals received the feed formulated for the kits, the difference in feed intake levels disappeared. It was previously demonstrated that this behavior led to a higher weaning weight with no negative effect on kit health (Read et al., 2016). An explanation could be the higher appeal of starch, as the energy in the mothers feed was supplied in the form of starch. In experiments of early weaning, high levels of starch were linked with higher growth in kits (Gidenne and Fortun-Lamothe, 2002). In our study, feed offering the highest energy levels had also the highest starch content. Further studies are necessary to get insight on feed preference at the onset of solid feed intake in rabbits, especially between energy levels and starch to promote early solid feed intake.

Unfortunately, in our study the nature of the diet at the onset of feeding and afterward had only limited effect on the cecal bacterial community. In human provided with diets varying in starch content or with reduced carbohydrates, the 16S rRNA sequences were reported to cluster more strongly by individual than by diet (Walker et al., 2011). In our study, inter-individual variations might have overtaken diet effects on microbial community.

Bacterial community of the animals that had access to low energy and protein and high fiber feed seemed to reach maturity faster; their communities were more similar to those observed in 49-day-old rabbits. One could suggest that higher maturity might be linked with lower incidence of digestive trouble. Digestive troubles occur in rabbits mostly around weaning when age related changes in bacterial community composition are high with a sharp decrease in *Bacteroides* in favor of members of Lachnospiraceae and Ruminococcaceae families, the latter being the most abundant in the ecosystem after weaning. Although no causal effect could clearly be demonstrated, two distinct studies reported that occurrence of epizootic rabbit enteropathy was correlated to the overgrowth of species in the *Bacteroides* genus (Bauerl et al., 2014; Jin et al., 2018). This reinforce the importance of the role of the age-related ecological succession of species that takes place in gut ecosystem as determinant of host health and disease. However, in the present study, the high sanitary status of the animals did not permit to determine a direct relationship between health preservation and microbiota evolution stability.

## Conclusion

In young rabbit, the bacterial communities evolve with age, showing a gradual increase in alpha-diversity and in bacterial relationship network complexity over time. An exclusively milk based diet constrains the bacterial community to a highly homogenous community structure but at the same time leads to high phylogenetic diversity. Once solid feed ingestion starts, a great shift in the structure and composition of the cecal bacterial community is observed, but a network of consistent interactions among bacterial species occurs only when solid feed intake is well established. At an early age, access to a high energy and protein feed stimulates feed intake, yet ingestion of a high fiber diet, at the expense of protein and energy, was found to accelerate the maturation of the bacterial community. However, this result needs to be confirmed with a more contrasted diet. Further studies are necessary to get an insight on nutritional factors responsible for stimulating feed intake and determine which nutrients have a positive action on colonization and maturation of the digestive ecosystem.

## Author Contributions

TR, LF-L, ND, TG, and SC conceived and designed the experiments. TR, SC, BG, CB, EB, OB, and LC performed the experiments. TR, SC, LC, MLB, and GP analyzed the data. TR, SC, and LF-L wrote the manuscript.

## Conflict of Interest Statement

The authors declare that the research was conducted in the absence of any commercial or financial relationships that could be construed as a potential conflict of interest.

## Footnotes

1 https://github.com/maloleboulch/MACADAMExplore

2 macadam.toulouse.inra.fr
