# Supplementary material for: Diversity and Co-occurrence Pattern Analysis of Cecal Microbiota Establishment at the Onset of Solid Feeding in Young Rabbits
Source: Front Microbiol. 2019 May 10;10:973. doi: 10.3389/fmicb.2019.00973 (PMC6524096; doi:10.3389/fmicb.2019.00973)
Supplement: Supplementary file 1 [file Data_Sheet_1.docx]

Supplementary Material

Diversity and co-occurrence pattern analysis of cecal microbiota establishment in young rabbits

Tehya Read^1,3^, Laurence Fortun-Lamothe^1^, Géraldine Pascal^1^, Malo Le Boulch^1^, Laurent Cauquil^1^, Beatrice. Gabinaud^1^, Carole Bannelier^1^, Elodie Balmisse^2^, Nicolas Destombes^3^, Olivier Bouchez^4^, Thierry Gidenne^1^, Sylvie Combes^1*^

*** Correspondence:** Sylvie Combes sylvie.combes@inra.fr

# Supplementary Figures and Tables

## Supplementary Figures


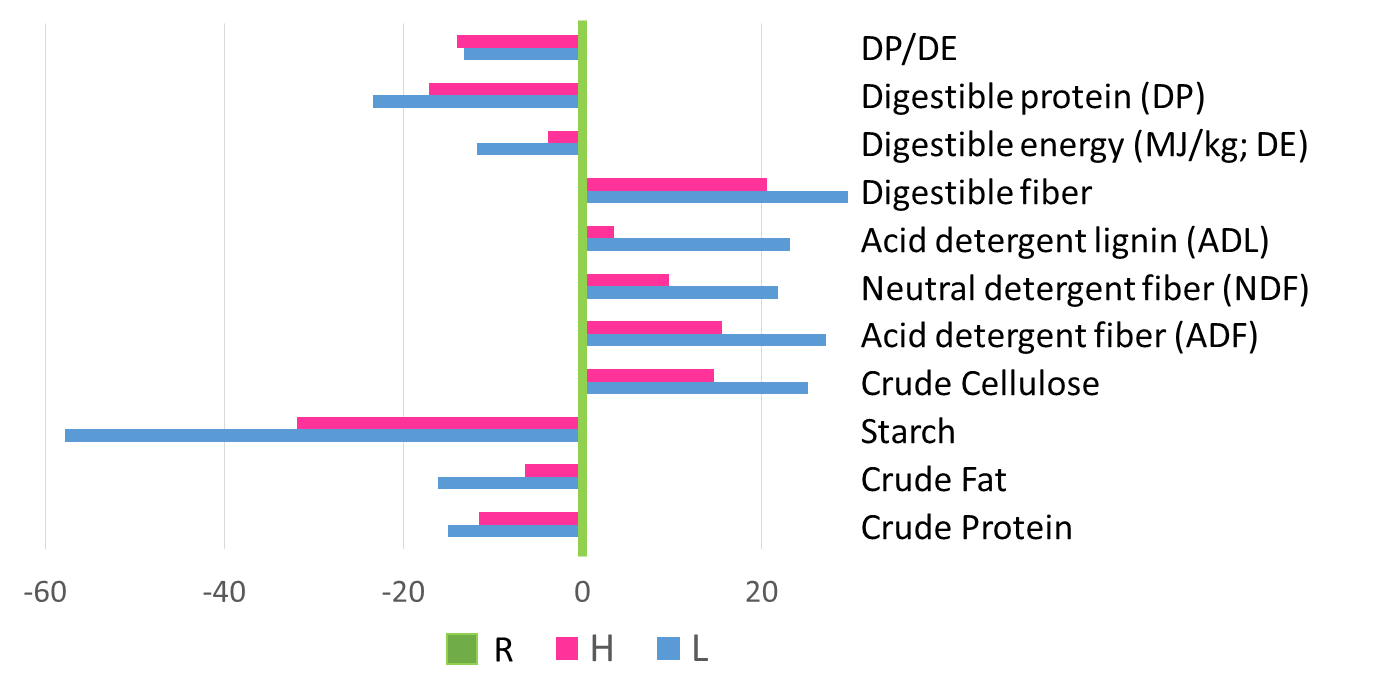


**Supplementary figure S1**: Illustration of chemical composition differences of high concentrate (H) and low concentrate (L) diets compared to the reproductive female diet (R) (percentage difference).


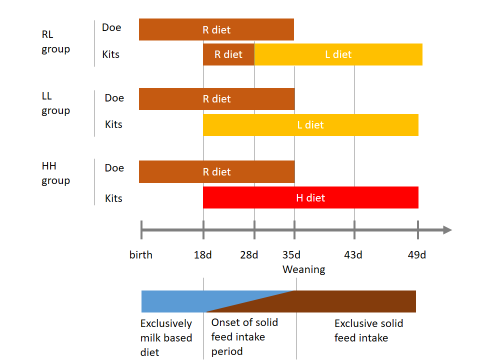


**Supplementary Figure S2.** Experimental design of the feeding strategy of rabbit kits and does. RL group. RL feeding strategy is close to current practices in French commercial rabbit farms whilst LL and HH feeding strategies were specifically designed for young rabbits. We hypothesized that these latter feeding strategies would be better adapted to promote health and growth of young rabbits

L (Low concentrate) and H (High concentrate) diets formulated to meet the needs of growing rabbits with differing energy and protein levels, and a constant DP/DE ratio

R (Reproduction diet): diet formulated to meet the needs of reproductive females


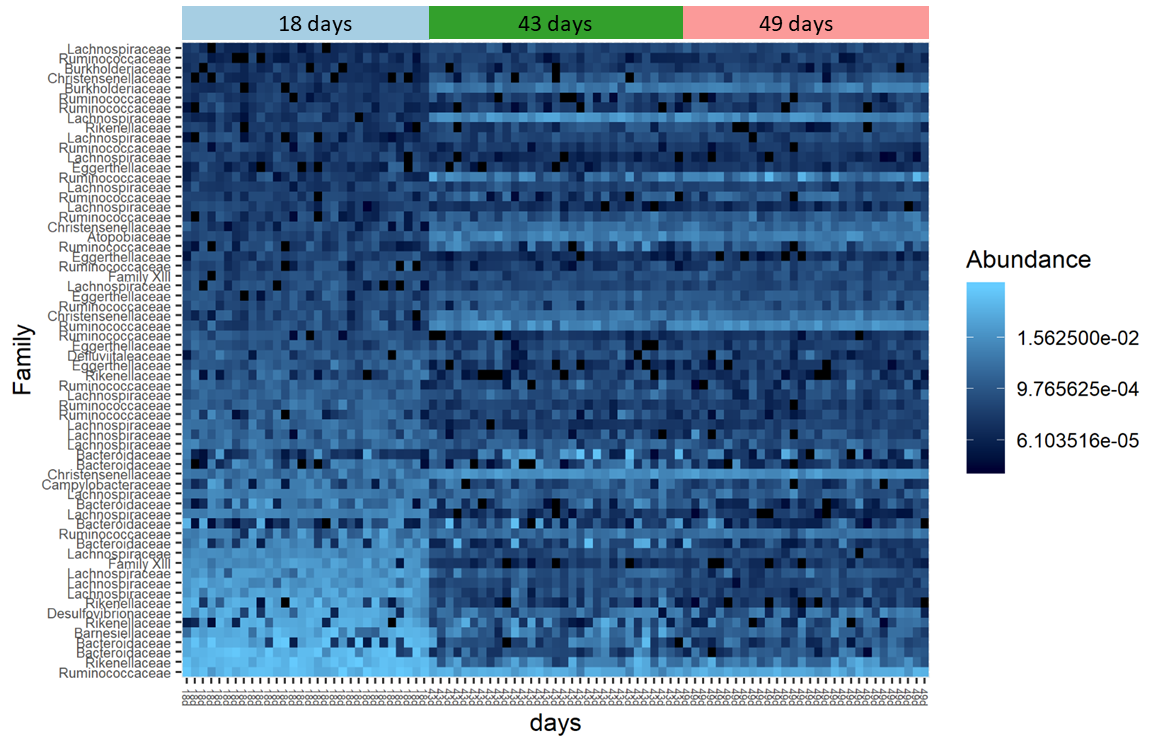


**Supplementary Figure S3.** Relative abundance of the 64 OTUs shared by exclusively milk-fed rabbits and their exclusively plant-based, solid feed 43 and 49 day-old counterparts. OTUs were labelled according to their family levels assignation. Only common OTUs present in 75% of individuals per age group are displayed

The 64 OTUs represent a relative abundance of 80.1%, 27.7% and 20.6% of the rabbit cecal bacterial community at 18 days, 43 days and 49 days respectively.

A.


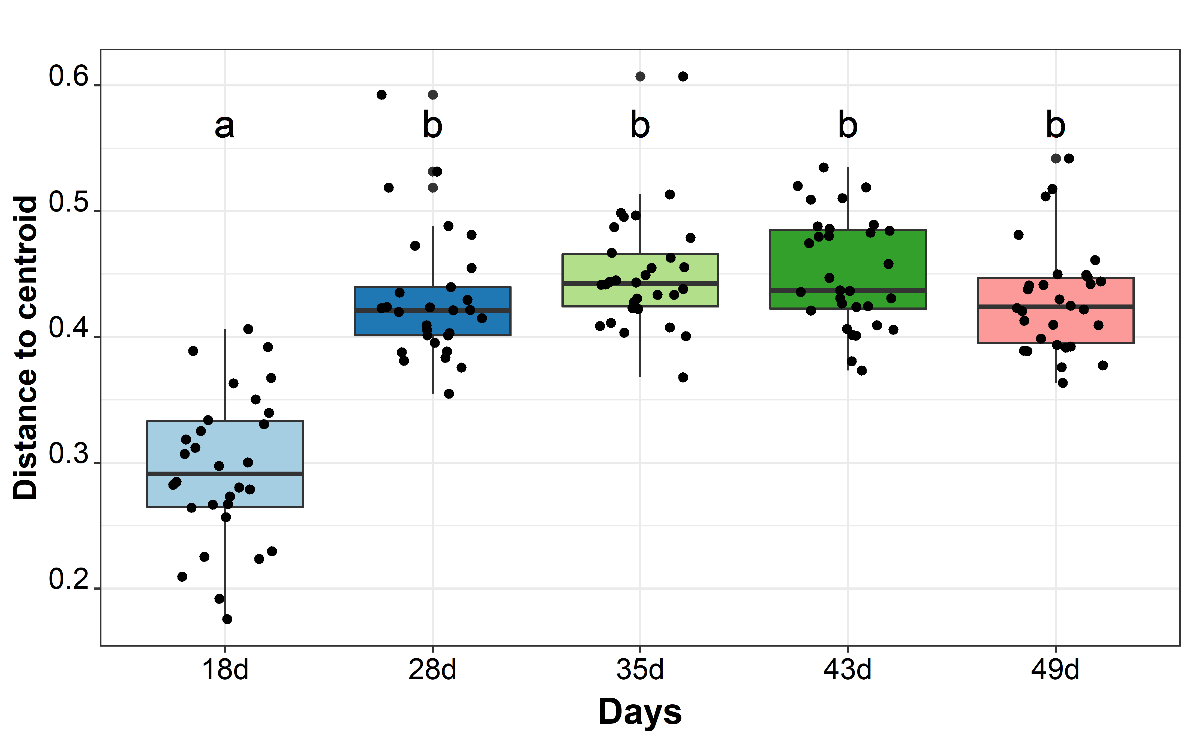


B.


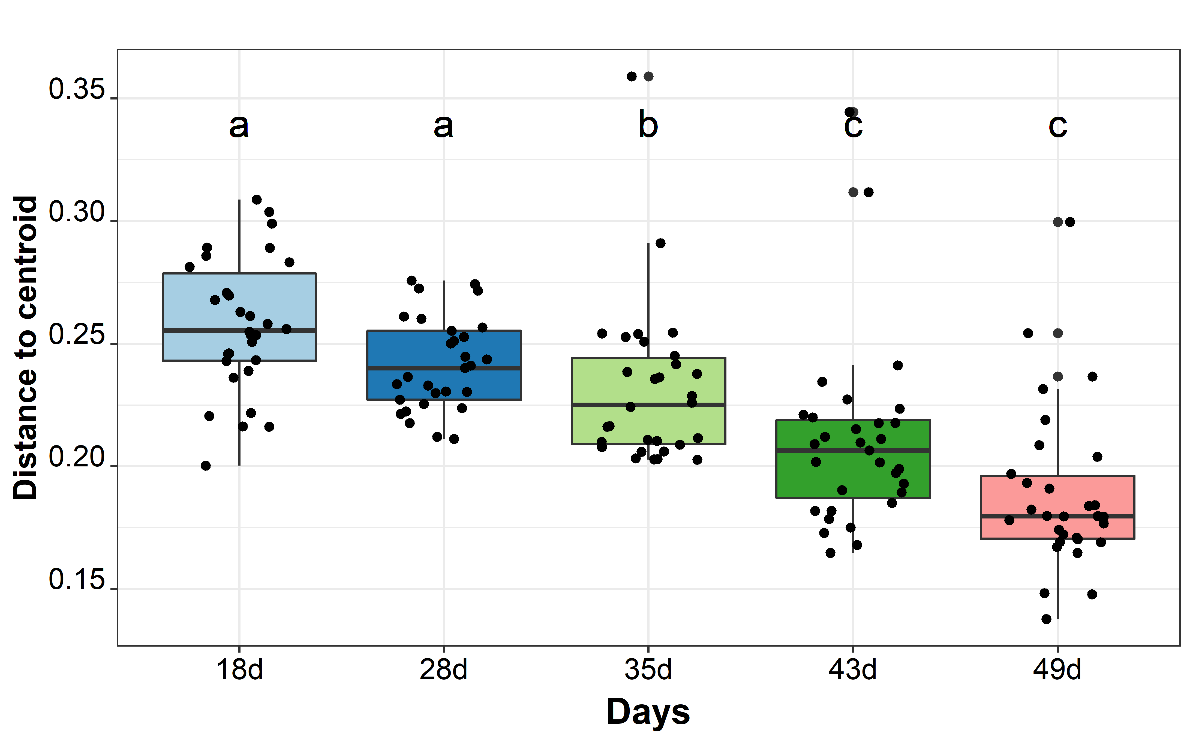


**Supplementary Figure S4** Cecal bacterial community within group dispersion as assessed by distance to centroid in Bray Curtis (A) and UniFrac (B) distance matrix


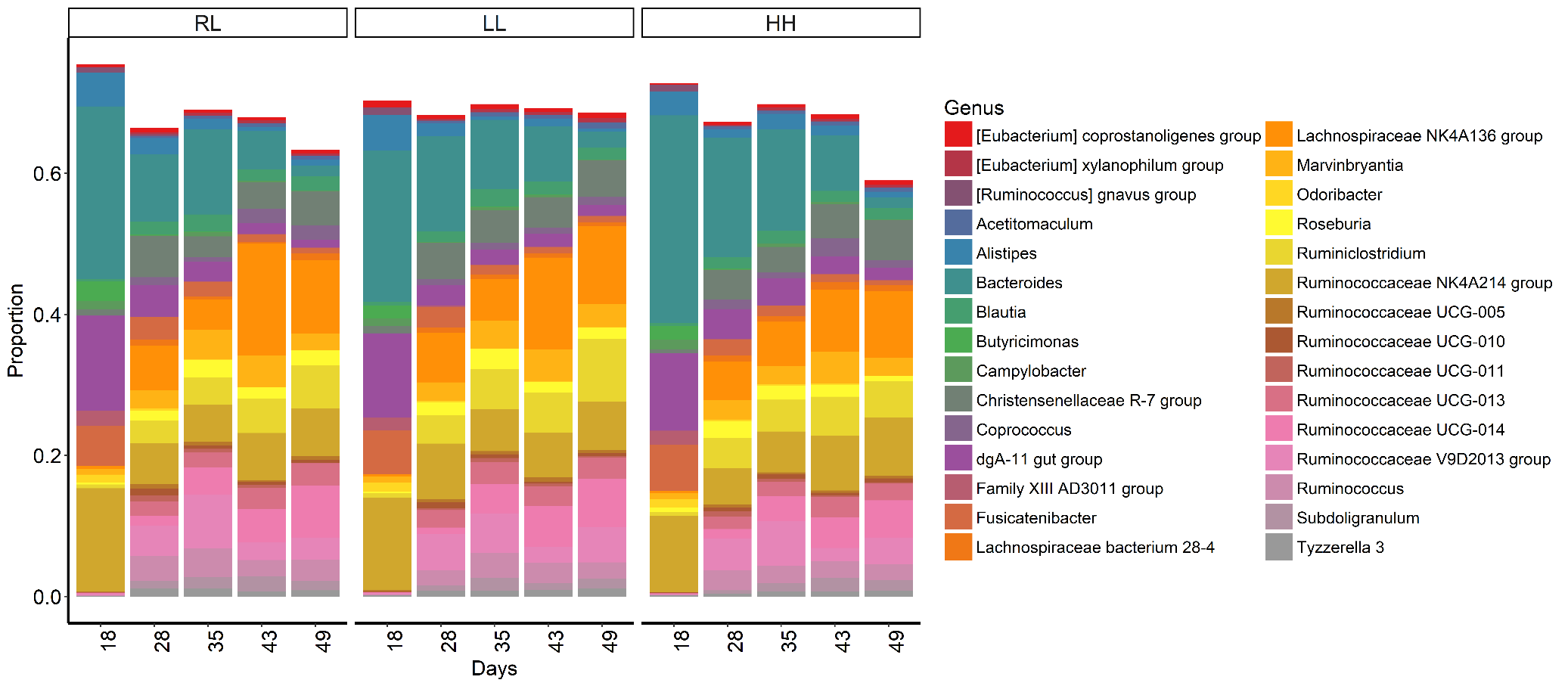


**Supplementary Figure S5** Age related changes of genus distribution in rabbit cecal bacterial communities in the 3 experimental groups

| A. | B. |
| --- | --- |
| 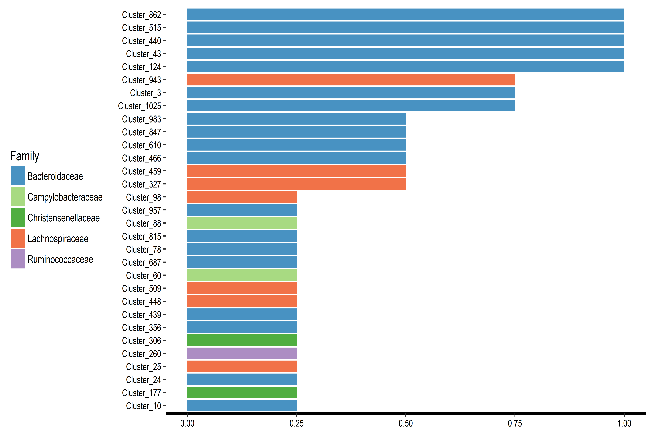 | 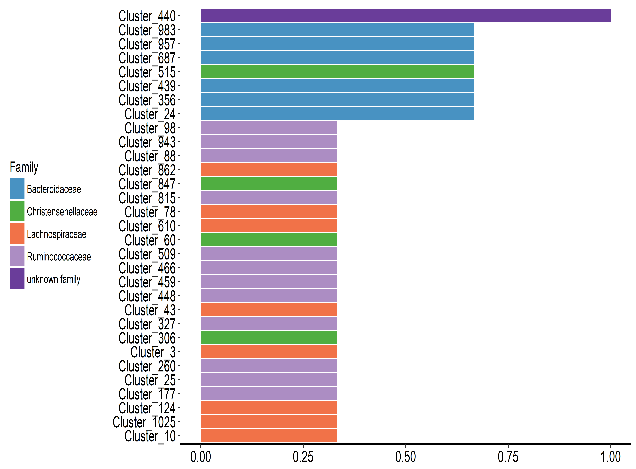 |
| C. | D. |
| 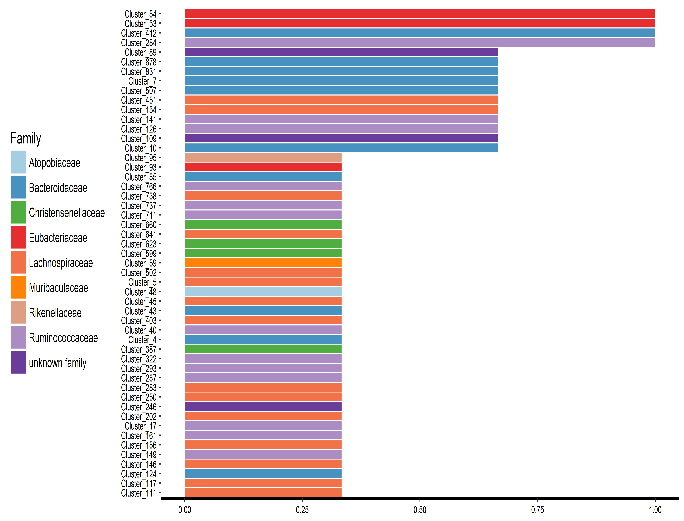 | 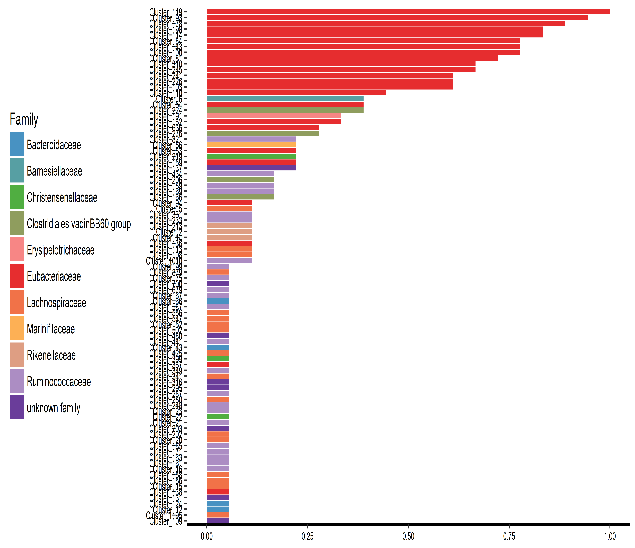 |
| E. |  |
| 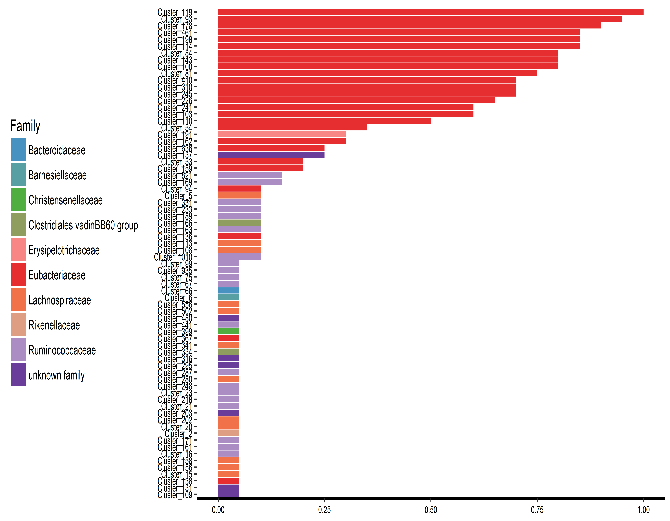 | 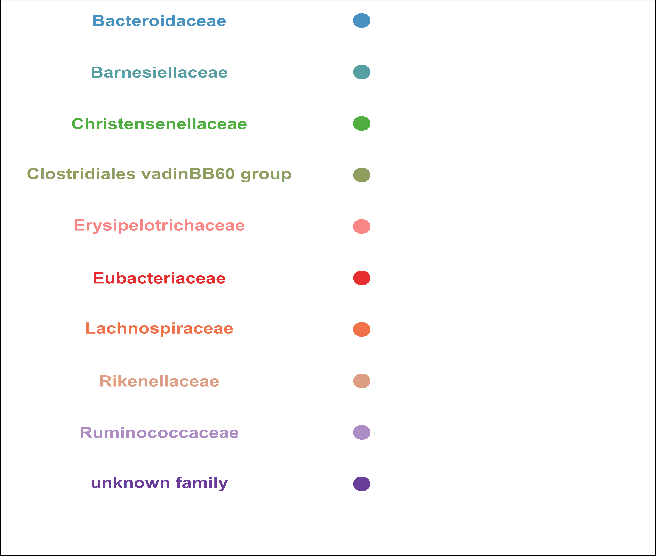 |

**Supplementary Figure S6** Number of degree for each node (OTU) in network of co-occurring bacterial OTUs in cecal microbiota for 18 (A), 28 (B), 35 (C), 43 (D) and 49 (E) day-old rabbits. The nodes were colored by family.

| A | B |
| --- | --- |
| 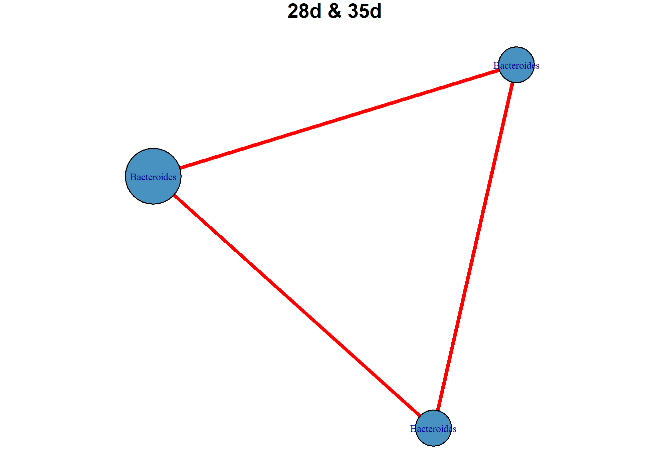 | 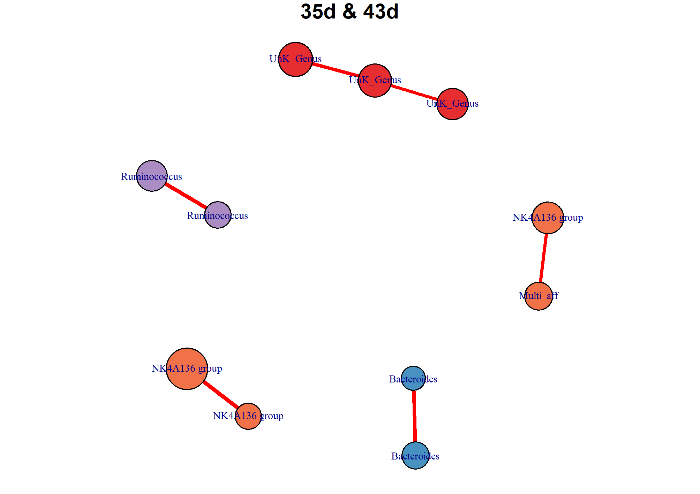 |
| C |  |
| 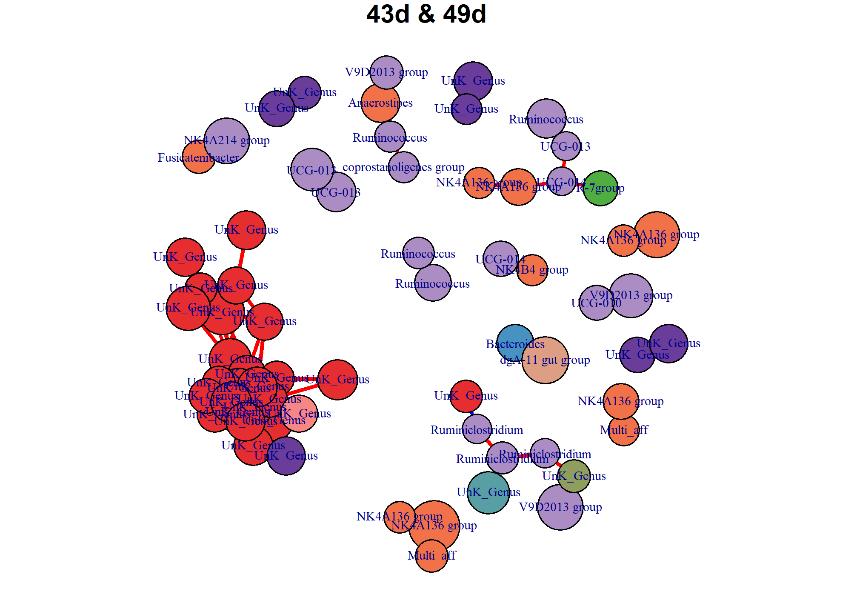 | 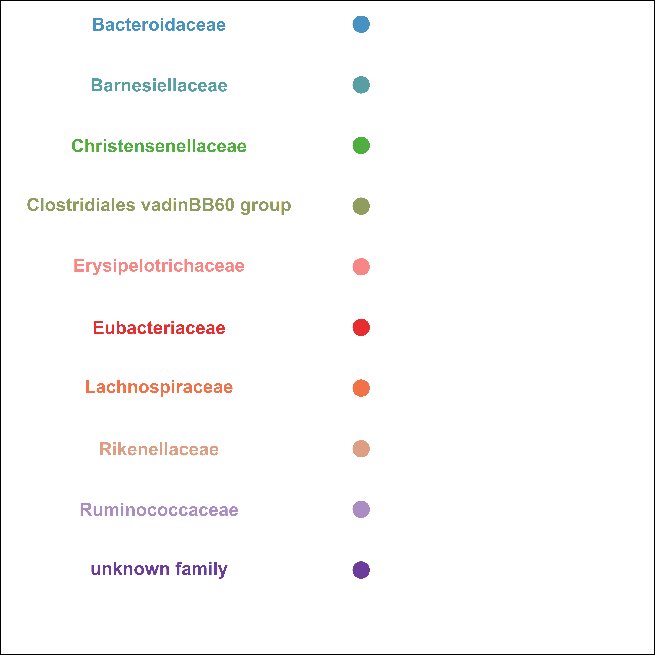 |

**Supplementary Figure S7:** Consistence of co-occurrence patterns across ecosystems from consecutive ages (A) between 28 and 35 days of age (A), between 35 and 43 days of age (B) and between 43 and 49 days of age (C).

No consistence of co-occurrence patterns could be observed between 18 and 28 days of age.


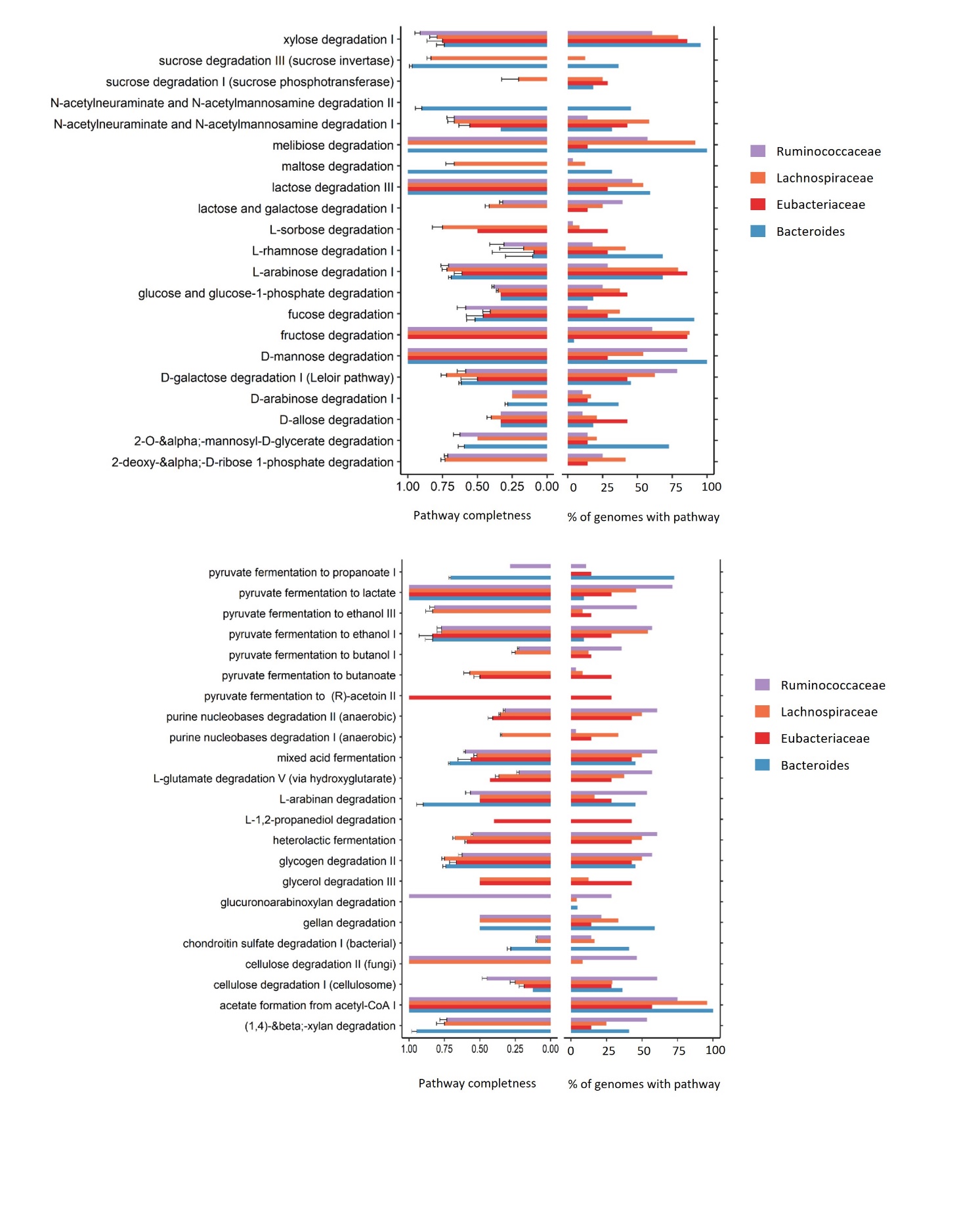


**Supplementary Figure S8:** Breakdown of carbohydrate (A) and polymeric compound degradation and fermentation (B) showing completeness of the pathways (left) and their percentages of presence in genomes (right) in Lachnospiraceae (n=24 genomes), Ruminococcaceae (n=28 genomes) and Eubacteriaceae (n=7 genomes) families and *Bacteroides* genus (n=22 genomes)

## Supplementary Tables

Table S1: Effect of the feeding strategy of young rabbits before weaning on growth performance

|  | 18 days | 28 days | 35 days | 43 days | 49 days |
| --- | --- | --- | --- | --- | --- |
| RL | 297±50 ^a^ | 554 ±92 | 877±123 ^b^ | 1158±134 ^b^ | 1405±156 ^b^ |
| LL | 309±52 ^b^ | 536±91 | 834±131 ^a^ | 1098±156 ^a^ | 1329±181 ^a^ |
| HH | 309±49 ^b^ | 545±88 | 854 ±110 ^ab^ | 1157±135 ^b^ | 1415±159 ^b^ |
| P value | 0.026 | 0.185 | 0.011 | <0.001 | <0.001 |

Table S2 Percentage (%) of phyla in cecal microbiota of young rabbits from 18 to 49 days of age.

|  | 18 days | 28 days | 35 days | 43 days | 49 days | Age | Diet Group | AgexGroup |
| --- | --- | --- | --- | --- | --- | --- | --- | --- |
| Actinobacteria | 0.58±0.27 ^a^ | 0.42±0.21 ^a^ | 1.2±0.7 ^b^ | 1.4±0.7 ^b^ | 1.2±0.5 ^b^ | <0.001 | 0.878 | 0.313 |
| Bacteroidetes | 55.8±7.7 ^d^ | 26.2±8.7 ^c^ | 21.1±13.2 ^c^ | 13.2±10.5 ^b^ | 5.7±4.5 ^a^ | <0.001 | 0.139 | 0.661 |
| Epsilonbacteraeota | 1.2±0.7 ^d^ | 0.09±0.12 ^a^ | 0.52±0.34 ^c^ | 0.29±0.29 ^b^ | 0.13±0.14 ^a^ | <0.001 | 0.496 | 0.342 |
| Firmicutes | 37.4±7.5 ^a^ | 71.9±8.9 ^b^ | 75.6±13.9 ^b^ | 83.5±10.7 ^c^ | 91.5±4.9 ^c^ | <0.001 | **0.039*** | 0.449 |
| Proteobacteria | 4.9±2.4 ^b^ | 1.3±0.7 ^a^ | 1.2±1.0 ^a^ | 1.5±0.6 ^a^ | 1.5±0.6 ^a^ | <0.001 | 0.547 | 0.370 |
| Tenericutes | 0.021±0.037 ^ab^ | 0.14±0.31 ^ab^ | 0.37±1.16 ^b^ | 0.016±0.072 ^a^ | 0.002±0.005 ^a^ | <0.001 | 0.117 | 0.450 |

* p value adjusted using BH test: 0.234

^a, b, c^ Means with different superscripts differ at *p* < 0.05

Table S3 Percentage (%) of main families in cecal microbiota of young rabbits from 18 to 49 days of age.

|  | | 18 days | 28 days | | | 35 days | | 43 days | 49 days | Age | Diet Group | | Agexgroup | |
| --- | --- | --- | --- | --- | --- | --- | --- | --- | --- | --- | --- | --- | --- | --- |
| **Bacteroidaceae** | | 25.5±7.8 ^d^ | 13.3±8.2 ^c^ | | | 12.2±11.6 ^c^ | | 7.1±7.3 ^b^ | 1.8±1.9 ^a^ | <0.001 | 0.404 | | 0.692 | |
| **Barnesiellaceae** | | 5.6±4.1 ^d^ | 3.6±2.2 ^cd^ | | | 2.2±1.7 ^c^ | | 1.0±1.6 ^b^ | 0.21±0.31 ^a^ | <0.001 | 0.148 | | 0.534 | |
| **Marinifilaceae** | | 3.4±1.3 ^c^ | 0.34±0.31 ^b^ | | | 0.18±0.15 ^b^ | | 0.11±0.14 ^a^ | 0.04±0.03 ^a^ | <0.001 | 0.277 | | 0.334 | |
| Muribaculaceae | | 0.18±0.36^a^ | 0.60±0.90^b^ | | | 0.66±1.71^b^ | | 0.73±1.54^b^ | 0.72±2.12^ab^ | <0.001 | 0.257 | | 0.005 | |
| **Rikenellaceae** | | 21.0±4.9 ^d^ | 8.2±3.8 ^c^ | | | 5.6±4.8 ^b^ | | 4.1±3.6 ^ab^ | 2.5±2.8 ^a^ | <0.001 | 0.312 | | 0.127 | |
| Burkholderiaceae | 0.041±0.019 ^a^ | | | 0.21±0.12 ^b^ | 0.41±0.29 ^c^ | | | 0.60±0.32 ^d^ | 0.70±0.34 ^d^ | <0.001 | 0.252 | | 0.659 | |
| **Campylobacteraceae** | | 1.21±0.68 ^d^ | 0.08±0.12 ^a^ | | | 0.52±0.34 ^c^ | | 0.29±0.29 ^b^ | 0.13±0.14 ^a^ | <0.001 | 0.496 | | 0.342 | |
| **Christensenellaceae** | | 0.92±0.68 ^a^ | 5.9±2.9 ^bc^ | | | 4.6±2.3 ^b^ | | 5.2±2.1 ^bc^ | 6.3±2.1 ^c^ | <0.001 | 0.062 | | **0.042** | |
| Clostridiales vadinBB60 group | | 0.46±0.60 ^a^ | 1.48±0.80 ^b^ | | | 0.94±0.60 ^b^ | | 0.53±0.75 ^a^ | 0.29±0.28 ^a^ | <0.001 | 0.920 | | 0.970 | |
| **Eubacteriaceae** | | 0.081±0.070 ^ab^ | 0.11±0.22 ^a^ | | | 0.60±1.32 ^b^ | | 5.39±5.6 ^c^ | 11.1±6.2 ^d^ | <0.001 | 0.471 | | **0.058** | |
| **Clostridiales FamilyXIII** | | 2.4±1.1 ^c^ | 0.18±0.19 ^a^ | | | 0.14±0.07 ^a^ | | 0.20±0.07 ^ab^ | 0.23±0.08 ^b^ | <0.001 | 0.819 | | 0.872 | |
| **Lachnospiraceae** | | 16.1±2.8 ^a^ | 27.1±4.8^b^ | | | 27.1±4.9^b^ | | 32.1±9.2 ^b^ | 28.4±7.9 ^b^ | <0.001 | 0.245 | | 0.133 | |
| **Ruminococcaceae** | | 17.1±6.6^a^ | 36.8±6.4^b^ | | | 41.6±9.7^b^ | | 37.4±7.8 ^b^ | 41.7±7.6 ^b^ | <0.001 | **0.022*** | | 0.914 | |
| Atopobiaceae | | 0.052±0.03^a^ | 0.11±0.11^a^ | | | 0.65±0.58^b^ | | 0.92±0.59 ^c^ | 0.75±0.42 ^bc^ | <0.001 | 0.358 | | 0.364 | |
| Eggerthellaceae | | 0.49±0.26^b^ | 0.30±0.13^a^ | | | 0.54±0.24^b^ | | 0.47±0.25 ^b^ | 0.43±0.14 ^b^ | <0.001 | 0.624 | | 0.157 | |
| **Desulfovibrionaceae** | | 4.9±2.4 c | 0.96±0.56 ^b^ | | | 0.55±0.56 ^a^ | | 0.83±0.40 ^b^ | 0.78±0.34 ^ab^ | <0.001 | 0.673 | | 0.155 | |
| Erysipelotrichaceae | | 0.002±0.002 ^a^ | 0.002±0.006 ^a^ | | | 0.007±0.024 ^a^ | 0.18±0.31 ^b^ | | 0.44±0.5 ^c^ | <0.001 | 0.162 | 0.316 | |  |
| Flavobacteriaceae | | 0.001±0.001 ^a^ | 0.048±0.09 ^ab^ | | | 0.060±0.162 ^ab^ | | 0.16±0.32 ^b^ | 0.34±0.80 ^b^ | <0.001 | 0.495 | | 0.647 | |

* p value adjusted using BH test 0.506

^a, b, c, d^ Means with different superscripts differ at p < 0.05

Table S4 Percentage (%) of main genera in cecal microbiota of young rabbits from 18 to 49 days of age

|  | 18 days | 28 days | 35 days | 43 days | 49 days | Age | Diet Group | Agexgroup |
| --- | --- | --- | --- | --- | --- | --- | --- | --- |
| Bacteroidaceae |  |  |  |  |  |  |  |  |
| ***Bacteroides*** | 25.1±7.6 ^d^ | 13.1±8.1 ^c^ | 12.1±11.5 ^c^ | 7.1±7.3 ^b^ | 1.8±1.8 ^a^ | <0.001 | 0.348 | 0.684 |
| Marinifilaceae |  |  |  |  |  |  |  |  |
| *Butyricimonas* | 2.2±1.5 | 0.093±0.1 | 0.048±0.068 | 0.031±0.051 | 0.018±0.020 | <0.001 | 0.693 | 0.167 |
| *Odoribacter* | 1.2±0.8 | 0.24±0.26 | 0.13±0.16 | 0.08±0.12 | 0.022±0.020 | <0.001 | 0.251 | 0.098 |
| Rikenellaceae |  |  |  |  |  |  |  |  |
| ***Alistipes*** | 4.4±3.1 ^c^ | 1.8±1.4 ^b^ | 1.3±1.7 ^ab^ | 1.0±1.1 ^a^ | 0.68±1.16 ^a^ | <0.001 | 0.561 | 0.064 |
| ***dgA-11 gut group*** | 12.1±4.2 ^c^ | 4.0±2.1 ^b^ | 2.9±2.5 ^b^ | 2.0±2.6^a^ | 1.4±1.9 ^a^ | <0.001 | 0.187 | 0.444 |
| Campylobacteraceae |  |  |  |  |  |  |  |  |
| *Campylobacter* | 1.2±0.7 | 0.088±0.123 | 0.52±0.34 | 0.28±0.29 | 0.13±0.14 | <0.001 | 0.495 | 0.342 |
| Christensenellaceae |  |  |  |  |  |  |  |  |
| ***R-7 group*** | 0.78±0.51 ^a^ | 5.08±2.20 ^c^ | 3.73±1.88 ^b^ | 4.31±1.82 ^bc^ | 5.20±1.98 ^c^ | <0.001 | 0.187 | 0.078 |
| Family XIII |  |  |  |  |  |  |  |  |
| *AD3011 group* | 1.9±1.2 | 0.094±0.171 | 0.038±0.023 | 0.084±0.039 | 0.089±0.064 | <0.001 | 0.949 | 0.334 |
| Lachnospiraceae |  |  |  |  |  |  |  |  |
| *[Eubacterium] xylanophilum group* | 0.003±0.006 | 0.20±0.23 | 0.44±0.38 | 0.38±0.34 | 0.43±0.46 | <0.001 | 0.725 | 0.454 |
| *[Ruminococcus] gnavus group* | 0.89±0.36 | 0.17±0.20 | 0.047±0.057 | 0.030±0.037 | 0.024±0.038 | <0.001 | 0.304 | 0.056 |
| *Acetitomaculum* | 0.002±0.003 | 0.31±0.21 | 0.49±0.26 | 0.53±0.21 | 0.69±0.35 | <0.001 | 0.123 | 0.008 |
| ***Blautia*** | 0.41±0.28 ^a^ | 1.70±0.83 ^b^ | 2.25±0.91 ^c^ | 1.66±0.61 ^bc^ | 1.76±0.68 ^bc^ | <0.001 | 0.614 | 0.625 |
| ***Coprococcus*** | 0.056±0.056 ^a^ | 1.1±1.2 ^b^ | 0.82±0.84 ^b^ | 1.8±2.7 ^b^ | 1.4±2.3 ^b^ | <0.001 | 0.917 | 0.394 |
| ***Fusicatenibacter*** | 6.2±2.4 ^d^ | 2.8±1.7 ^c^ | 1.6±1.0 ^b^ | 1.0±0.4 ^a^ | 0.77±0.44 ^a^ | <0.001 | 0.904 | 0.371 |
| *bacterium 28-4* | 0.013±0.022 | 0.83±0.85 | 0.62±0.72 | 0.68±1.26 | 0.80±0.80 | <0.001 | 0.291 | 0.612 |
| ***NK4A136 group*** | 0.37±0.25 ^a^ | 6.2±3.9 ^bc^ | 5.5±4.6 ^c^ | 12.4±9.8 ^d^ | 10.3±7.2 ^cd^ | <0.001 | 0.515 | 0.296 |
| ***Marvinbryantia*** | 0.83±0.32 ^a^ | 2.7±1.9 ^b^ | 3.6±2.0 ^bc^ | 4.5±2.3 ^c^ | 2.7±1.2 ^b^ | <0.001 | 0.552 | 0.632 |
| ***Roseburia*** | 0.37±0.57 ^a^ | 1.8±1.1 ^bc^ | 2.4±1.2 ^c^ | 1.6±0.9 ^bc^ | 1.5±1.1 ^b^ | <0.001 | 0.619 | 0.0001 |
| *Tyzzerella* | 0.12±0.18 | 0.81±0.65 | 0.91±0.73 | 0.78±0.51 | 0.98±0.86 | <0.001 | 0.262 | 0.217 |
| Ruminococcaceae |  |  |  |  |  |  |  |  |
| *[Eubacterium] coprostanoligenes group* | 0.53±0.99 | 0.57±0.31 | 0.52±0.36 | 0.55±0.32 | 0.65±0.33 | <0.001 | 0.563 | 0.402 |
| ***Ruminiclostridium*** | 0.54±0.17 ^a^ | 3.8±2.2 ^b^ | 4.7±3.0 ^bc^ | 5.3±3.2 ^bc^ | 6.7±5.0 ^c^ | <0.001 | 0.241 | 0.721 |
| ***NK4A214 group*** | 12.9±6.2 ^b^ | 6.1±2.9 ^a^ | 5.6±2.4 ^a^ | 7.0±3.1 ^a^ | 7.3±2.5 ^a^ | <0.001 | 0.870 | 0.338 |
| *UCG-005 group* | 0.099±0.27 | 0.52±0.47 | 0.43±0.30 | 0.40±0.33 | 0.41±0.39 | <0.001 | 0.053 | 0.011 |
| *UCG-010 group* | 0.049±0.025 | 0.79±0.57 | 0.54±0.41 | 0.36±0.36 | 0.49±0.43 | <0.001 | 0.705 | 0.604 |
| *UCG-011 group* | 0.066±0.11 | 0.64±0.63 | 0.52±0.54 | 0.30±0.40 | 0.13±0.16 | <0.001 | 0.872 | 0.727 |
| ***UCG-013 group*** | 0.033±0.029 ^a^ | 2.1±0.8 ^b^ | 2.4±1.1 ^bc^ | 2.9±1.0 ^c^ | 2.8±1.2 ^bc^ | <0.001 | 0.071 | 0.634 |
| ***UCG-014 group*** | 0.059±0.05 ^a^ | 1.3±0.95 ^b^ | 3.8±2.9 ^c^ | 4.9±2.9 ^cd^ | 6.5±2.4 ^d^ | <0.001 | 0.594 | 0.776 |
| ***V9D2013 group*** | 0.21±0.17 ^a^ | 4.6±3.1 ^cd^ | 6.5±3.5 ^d^ | 2.2±2.1 ^b^ | 3.9±3.7 ^c^ | <0.001 | 0.352 | 0.844 |
| ***Ruminococcus*** | 0.15±0.12 ^a^ | 5.8±3.3 ^b^ | 6.6±3.6 ^b^ | 5.0±2.0 ^b^ | 5.0±1.9 ^b^ | <0.001 | 0.125 | 0.202 |
| ***Subdoligranulum*** | 0.024±0.019 ^a^ | 0.79±0.60 ^b^ | 1.5±0.8 ^c^ | 1.7±2.0 ^c^ | 1.4±0.9 ^c^ | <0.001 | 0.339 | 0.038 |

Table S5 Influence of the feeding strategy and age on cecal fermentation characteristics of young rabbits.

| **Age (days)** | **Diet Group** | **pH** | **Dry Matter, %** | **VFA total, mmol/L** | **Acetate, %** | **Propionate, %** | **Butyrate, %** | **Ratio, C3/C4^1^** | **NH3, mmol/L** |
| --- | --- | --- | --- | --- | --- | --- | --- | --- | --- |
| **18** | RL | 6.9 | 18.8 | 29.7 | 86.8 | 9.0 | 4.2 | 2.17 | NA |
|  | LL | 6.9 | 18.2 | 25.6 | 86.7 | 9.2 | 4.1 | 2.28 | NA |
|  | HH | 6.8 | 15.1 | 24.4 | 88.6 | 7.9 | 3.5 | 2.27 | NA |
| **28** | RL | 5.8 | 26.2 | 80.8 | 84.5 | 4.9 | 10.6 | 0.47 | 16.4 |
|  | LL | 5.8 | 22.7 | 80.9 | 83.2 | 5.9 | 10.9 | 0.54 | 9.53 |
|  | HH | 5.7 | 23.1 | 87.1 | 82.9 | 5.6 | 11.5 | 0.49 | 9.42 |
| **35** | RL | 5.7 | 24.7 | 84.6 | 81.8 | 5.8 | 12.4 | 0.46 | 18.9 |
|  | LL | 5.9 | 23.7 | 78.6 | 82.9 | 5.4 | 11.7 | 0.46 | 13.5 |
|  | HH | 5.9 | 24.4 | 66.5 | 80.7 | 6.2 | 13.1 | 0.48 | 15.1 |
| **42** | RL | 6.1 | 23.5 | 31.5 | 80.2 | 7.1 | 12.7 | 0.57 | 24.2 |
|  | LL | 6.1 | 24.1 | 31.9 | 80.5 | 6.9 | 12.6 | 0.55 | 23.6 |
|  | HH | 6.1 | 23.5 | 31.3 | 80.7 | 7.1 | 12.2 | 0.58 | 23.9 |
| **49** | RL | 6.2 | 21.3 | 30.7 | 79.2 | 7.3 | 13.5 | 0.54 | 26.7 |
|  | LL | 6.2 | 21.3 | 29.0 | 81.3 | 6.7 | 12.0 | 0.56 | 28.5 |
|  | HH | 6.3 | 20.2 | 29.3 | 80.1 | 6.9 | 13.0 | 0.53 | 23.7 |
| **SEM** |  | 0.1 | 0.3 | 2.3 | 1.9 | 0.1 | 0.3 | 0.06 | 0.6 |
| **P-value** | Age | <0.001 | <0.001 | <0.001 | <0.001 | <0.001 | <0.001 | <0.001 | <0.001 |
|  | Group | 0.264 | 0.091 | 0.187 | 0.404 | 0.899 | 0.711 | 0.885 | 0.003 |
|  | Age*Group | 0.229 | 0.021 | 0.167 | 0.786 | 0.185 | 0.766 | 0.984 | 0.006 |

^1^Ratio C3/C4: ratio propionate/butyrate.

Table S6 Influence of the feeding strategy and age on full cecum weight and proportion of full cecum weight relative to body weight of young rabbits.

| **Age (days)** | **Diet group** | **Full cecum weight (g)** | **Proportion of full cecum weight relative to body weight (%)** |
| --- | --- | --- | --- |
| 18 | RL | 5.5 | 1.78 |
|  | LL | 5.7 | 1.83 |
|  | HH | 5.6 | 1.79 |
| 28 | RL | 24.3 | 4.21b |
|  | LL | 23.8 | 4.76ab |
|  | HH | 26.7 | 5.20a |
| 35 | RL | 50.9 | 5.65 |
|  | LL | 50.6 | 6.04 |
|  | HH | 51.8 | 6.12 |
| 43 | RL | 76.6 | 7.27 |
|  | LL | 74.8 | 7.14 |
|  | HH | 73.6 | 6.88 |
| 49 | RL | 112.1 | 8.51ab |
|  | LL | 115.1 | 9.23 a |
|  | HH | 110.4 | 8.35 b |
| **SEM** |  | 3.1 | 0.20 |
| **P-value** | Age | <0.001 | <0.001 |
|  | Diet Group | 0.881 | 0.044 |
|  | Age*Diet group | 0.920 | 0.231 |
